# Correlation between the ablation ratio and posterior corneal stability after small incision lenticule extraction for high myopia

**DOI:** 10.1007/s00417-023-05979-5

**Published:** 2023-01-20

**Authors:** Liyuan Yang, Shengtao Liu, Xingtao Zhou, Yu Zhao

**Affiliations:** 1 Department of Ophthalmology, Gansu Provincial Hospital, Lanzhou, Gansu China; 2 Department of Ophthalmology and Optometry, Eye and ENT Hospital, Fudan University, 83 Fenyang Road, Shanghai, 200031 People’s Republic of China; 3 NHC Key Laboratory of Myopia (Fudan University), Laboratory of Myopia, Chinese Academy of Medical Sciences, Shanghai, China; 4 Shanghai Research Center of Ophthalmology and Optometry, Shanghai, China

**Keywords:** Ablation ratio, Posterior corneal stability, High myopia, SMILE

## Abstract

**Purpose:**

The aim of this study is to investigate changes in posterior corneal elevation and their correlations with the ablation ratio 3 years after small incision lenticule extraction (SMILE) for high myopia.

**Methods:**

Eighty eyes underwent SMILE were enrolled in this study. Eyes were classified into two groups based on the ablation ratio (AR, lenticule thickness from SMILE machine/thinnest corneal thickness): group A (< 25%, 40 eyes) and group B (≥ 25%, 40 eyes). Pentacam was used to measure the posterior corneal elevation at the central point, thinnest point, and posterior maximum elevation (PME) and the mean posterior elevation in the central 2-mm area (MPE-2 mm), 4-mm area, and 6-mm area at the 3-year follow-up.

**Results:**

More than 85% of the eyes had an AR of less than 27%, and no cases of iatrogenic keratectasia developed. In both groups, for central region, posterior elevation decreased implying backward displacement; for peripheral region, it increased indicating forward trend. There was no significant difference in changes in all determined parameters between the two groups (*P* ≥ 0.07). Moreover, no significant correlation was noted between AR and posterior elevation changes. In group A, decreasing changes in PME (*r* =  − 0.42, *P* = 0.01) and MPE-2 mm (*r* = 0.40, *P* = 0.01) demonstrated negative correlations with residual bed thickness.

**Conclusion:**

Region-dependent changes were demonstrated in the eyes that underwent SMILE. The central area showed a subtly declining posterior elevation, and the peripheral area showed a slightly increasing elevation. The limited ablation ratio had no impact on the changes in posterior corneal elevation.

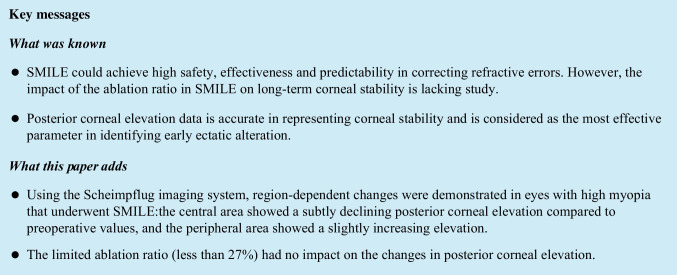

## Background

Small incision lenticule extraction (SMILE) has emerged as a flap-free corneal refractive surgery for more than 10 years since Sekundo and Shah et al. first reported it in 2011 [1, 2]. Unlike traditional corneal refractive surgery, it only requires a femtosecond laser to create an intra-stromal lenticule, followed by manual extraction of the lenticule through the small incision [3, 4]. Accumulating studies have documented that SMILE could achieve good predictability, high efficiency, and years of stability [1, 2, 5]. Presently, it has gained favorable acceptance from both eye surgeons and patients worldwide. However, the long-term safety of SMILE remains the first priority and should always be kept in mind in clinical practice [6].

Iatrogenic keratectasia, a severe complication following almost all kinds of corneal refractive surgery, is characterized by decreasing visual acuity, progressive corneal thinning, and corneal forward protrusion, and some extreme cases may need corneal transplantation [7]. Posterior corneal elevation has been confirmed as an effective indicator in diagnosing early-onset keratoconus [8, 9], and in terms of screening iatrogenic keratectasia, it has several advantages over other parameters, such as an absence of effects by tear film or alteration of the anterior corneal surface [10, 11].

Some studies have demonstrated that the posterior corneal surface shows no forward protrusion after SMILE [12, 13]. However, the effect of the ablation ratio (the percentage of tissue removed) in SMILE on the posterior corneal surface remains ambiguous. Whether the cornea in which thicker tissue was removed has an increased rate of instability and is prone to iatrogenic keratectasia needs to be further investigated.

In the present study, we aimed to evaluate the 3-year posterior corneal stability after SMILE due to high myopia and explore the correlation between the ablation ratio and posterior corneal elevation changes.

## Methods

### Patients

This study was performed in accordance with the tenets of the Declaration of Helsinki, and the Ethics Committee of Fudan University and the ENT Hospital Review board (Shanghai, China) approved the study protocol (KJ2008-10). All participants in the study were fully explained the procedure of refractive surgery and provided written informed consent.

Eighty eyes of 80 patients who underwent SMILE surgery at Eye and ENT Hospital of Fudan University, Shanghai, China from Jan 2019 to Mar 2019 were included in this retrospective study. One eye was randomly selected if both eyes of one patient met the study criteria. Inclusion criteria were as follows: (1) age > 18 years; (2) stable spherical equivalent, spherical equivalent growth ≤ 0.50 D in the past 2 years; (3) refractive error: sphere of − 6.00 DS to − 10.00 DS and astigmatism of less than − 4.00 DC; (4) absence of contact lens time: soft contact lenses for more than 2 weeks, rigid gas permeable contact lenses for > 1 month, and orthokeratology lens for ≥ 3 months; (5) minimal corneal thickness ≥ 480 µm. Exclusion criteria were as follows: (1) systemic or infectious diseases; (2) ocular inflammation, keratoconus, or severe dry eye; (3) an obvious corneal scar, corneal degeneration, corneal dystrophy, keratitis, cataract, retinal detachment, or other fundus diseases; and (4) any intraoperative or postoperative complications.

**Fig. 1:**
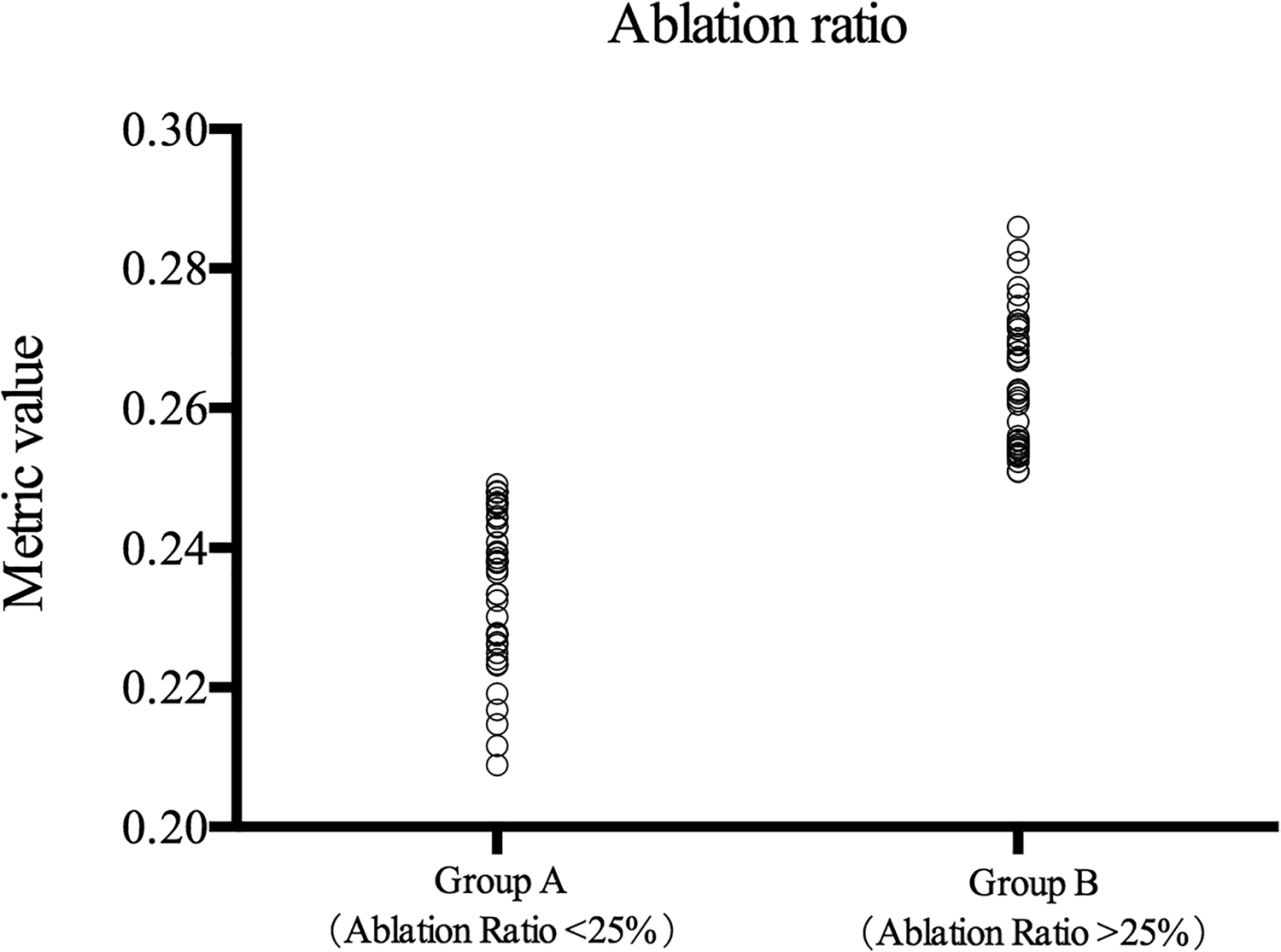
Scatter plot graph of all the eyes according to different ablation ration included in the study: group A (ablation ratio < 25%) and group B (ablation ratio > 25%)

According to the ablation ratio (AR, lenticule thickness from SMILE machine/thinnest corneal thickness), the eyes were divided into groups A (< 25%, 40 eyes) and B (≥ 25%, 40 eyes)(Fig. 1). All participants underwent a comprehensive preoperative examination, including slit-lamp examination, uncorrected distance visual acuity (UDVA), corrected distance visual acuity (CDVA), manifest refraction, intraocular pressure, and corneal topography by Pentacam HR imaging (Oculus GmbH, Wetzlar, Germany).

### SMILE procedures

All SMILE procedures were performed by one experienced surgeon (XZ) using the VisuMax femtosecond laser system (Carl Zeiss Meditec AG, Germany). The repetition rate and the pulse energy were set at 500 kHz and 130 nJ, respectively. The intended cap diameter and thickness were set at 7.5 mm and 100–120 μm, respectively. The optical zone varied from 6.0 to 6.8 mm according to the preoperative thinnest corneal thickness and programmed corrected refractive error. Patients had topical anesthesia instilled and were then positioned under the contact curve glass. Once corneal centration was guaranteed, the surgeon initiated the suction and began femtosecond laser application. The whole application phase lasted for half a minute to create an intra-stromal refractive lenticule and a small incision located at 90°. After completion of laser scanning, the surgeon dissected the lenticule interface, followed by extraction of the lenticule manually with precaution.

The postoperative medication regimen included the usage of topical 0.5% levofloxacin (Santen Pharmaceutical, Osaka, Japan) four times per day for 1 week, 0.1% fluorometholone eye drops (Santen Pharmaceutical, Osaka, Japan) from eight times to one time per day over 24 days in sequential decreasing order. Sodium hyaluronate (0.3%) eye drop was also used four times per day from 1 to 3 months as needed. All the eye drops were used simultaneously at 1 day postoperatively.

### Data collection

The lenticule thickness was extracted directly from the VisuMax system. Corneal tomography images were acquired using the Pentacam HR imaging system before and 3 years after SMILE. Images were acquired under the standard Pentacam instruction manual. Only measurements with “OK” statement were accepted and saved for further analysis. The reference best-fit-sphere (BFS) was defined in the central 8.0 mm corneal region [14]. To ensure that the BFS was identical between pre- and post-surgery images, elevation data were extracted from the compare-two-image model with BFS as the preoperative value.

Posterior central elevation (PCE), posterior elevation at the preoperative thinnest point (PTE), and posterior maximal elevation (PME) in the central 4.0 mm area above the BFS were recorded. Twenty-six points within the 6 mm central cornea (4 points: 1 mm from the center at 45°, 135°, 225°, and 315°; 8 points: 2 mm from the center at 0°, 45°, 90°, 135°, 180°, 225°, 270°, and 315°; 14 points: 3 mm from the center at 15°, 45°, 75°, 90°, 105°, 135°, 165°, 195°, 225°, 255°, 270°, 285°, 315°, and 345°) were also extracted. The average data of 2-mm central cornea area, 4-mm diameter central cornea area, and 6-mm central cornea area points were recorded as the mean posterior elevation (MPE-2 mm, MPE-4 mm, and MPE-6 mm, respectively). The change in elevation was defined as the postoperative data minus preoperative data, and a positive value was regarded as a forward shift.

### Statistical analysis

Statistical software SPSS ver.25.0 was used for statistical analysis. Data with normal distribution are expressed as mean ± standard deviation. The Kolmogorov–Smirnov method was used to test the normality of the data, followed by a test for homogeneity of variances. The analysis of variance test was used for categorical data with a normal distribution, and we used the Mann–Whitney *U* test for data with a non-normal distribution. For continuous variables with normal distribution, we used the Pearson correlation test to analyze the correlation between the AR and changes in posterior corneal elevation; if the data were not suitable for Pearson correlation test, the Spearman correlation test was used. A *P* value of < 0.05 was indicated as a statistically significant difference.

## Results

All surgeries were finished successfully. No complications occurred either during or after the procedures. Patients’ characteristics are presented in Table 1.

**Table 1:** Patient characteristics in two groups

	Group A	Group B	*P* value
Age (years)	29.68 ± 5.16 (20 to 44)	29.33 ± 5.42 (19 to 40)	0.43
Gender (male/female)	17/23	15/25	0.26
Preoperative SE (D)	− 6.83 ± 0.74 (− 6.00 to − 8.63)	− 7.42 ± 0.97 (− 6.25 to − 9.13)	0.01*
Preoperative CCT (μm)	534.7 ± 36.1 (488 to 632)	536.7 ± 31.2 (494 to 621)	0.67
AD (µm)	125.3 ± 8.1 (106 to 141)	141.6 ± 9.9 (126 to 158)	0.01*
RBT (µm)	294.7 ± 25.3 (261 to329)	283.7 ± 18.6 (263 to 316)	0.01*
AR	0.236 ± 0.011 (0.209 to 0.249)	0.263 ± 0.009 (0.251 to 0.282)	0.01*

*D* diopters; *SE* spherical equivalent, *CCT* thinnest central corneal thickness, *AD* ablation depth, *RBT* residual bed thickness, *AR* ablation ratio

^*^Difference is significant at the 0.05 level

### Visual outcomes

The safety index was 1.14 in group A and 1.13 in group B. The efficacy index was 1.08 in group A and 1.09 in group B. No statistical difference was found between the two groups. Ninety-eight percent of eyes in each group (39/40) achieved a UDVA of 20/20 or better, and no eye lost one or more lines of CDVA in either group. Forty-three percent (17/40) of the treated eyes in group A and 48% (19/40) in group B acquired one or more lines of CDVA.

**Fig. 2:**
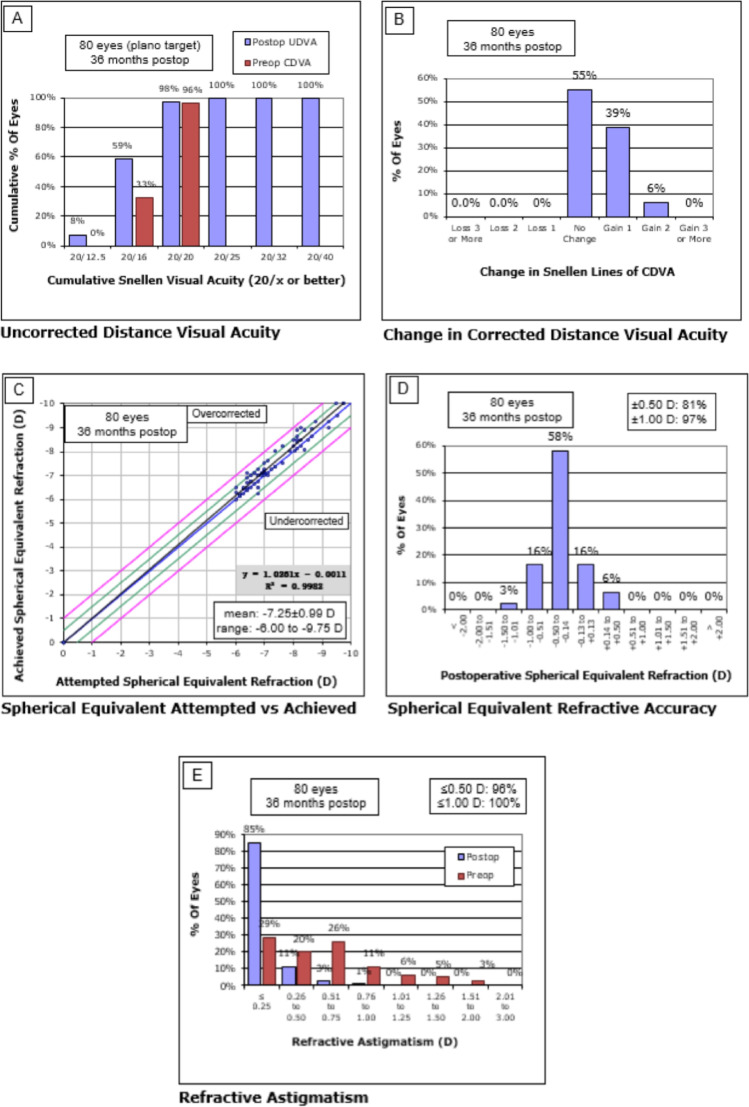
Refractive outcomes of 80 eyes 3 years after SMILE. *UDVA* uncorrected distance visual acuity, *CDVA* corrected distance visual acuity, *D* diopters, *Postop* postoperative, *Preop* preoperative. **A** Uncorrected distance visual acuity. **B** Change in corrected distance visual acuity. **C** Spherical equivalent attempted vs achieved. **D** Spherical equivalent refractive accuracy. **E** Refractive astigmatism

The mean spherical equivalent (SE) was − 0.32 ± 0.48 D in group A and − 0.34 ± 0.46 D in group B 3 years after SMILE. The percentages of eyes with postoperative SE within ± 0.5 D and ± 1.0 D were 81% and 98%, respectively; 83% (33/40) of the eyes in group A and 80% (32/40) in group B were within ± 0.5 D. Thirty-nine eyes in group A and 38 eyes in group B had stable astigmatism, with changes in diopters less than 0.50 (Fig. 2).

### Posterior corneal elevation

PCE and PTE significantly decreased 3 years after SMILE compared with that preoperatively, indicating no forward bulge (*P* ≤ 0.04). A slight backward change was also observed in PME and MPE-2 mm, but a statistically significant difference was only detected for PME in group A (*P* = 0.02). MPE-6 mm, which represents the posterior corneal surface in the peripheral area, increased from the baseline levels in group A (*P* = 0.02) and group B (*P* = 0.09). Data of posterior corneal elevation before and after SMILE in the two groups are described in Table 2.

**Table 2:** Posterior corneal elevation before and after SMILE in two groups

		Time point		*P* value			Time point		*P* value
		Preop	Postop 3y				Preop	Postop 3y	
Group A	PCE	1.31 ± 1.89	0.03 ± 2.60	0.02*	Group B	PCE	1.59 ± 2.33	0.38 ± 2.92	0.04*
	PTE	4.31 ± 2.34	0.69 ± 2.51	0.01*		PTE	3.77 ± 2.61	0.87 ± 3.01	0.01*
	PME	5.08 ± 1.83	4.02 ± 2.11	0.02*		PME	5.03 ± 2.02	4.23 ± 2.53	0.12
	MPE-2 mm	2.07 ± 1.35	0.85 ± 1.98	0.05*		MPE-2 mm	2.07 ± 1.58	1.21 ± 2.27	0.06
	MPE-4 mm	− 0.35 ± 1.64	0.06 ± 1.85	0.31		MPE-4 mm	− 0.21 ± 1.59	− 0.27 ± 1.81	0.87
	MPE-6 mm	− 5.78 ± 2.52	− 3.93 ± 2.63	0.02*		MPE-6 mm	− 5.12 ± 2.26	− 4.13 ± 2.88	0.09

*PCE* posterior central elevation, *PTE* posterior thinnest elevation, *PME* posterior maximal elevation, *MPE*-2 mm mean posterior elevation in the central 2 mm region, *MPE*-4 mm mean posterior elevation in the central 4 mm region, *MPE*-6 mm mean posterior elevation in the central 6 mm region

^*^Difference is significant at the 0.05 level

**Fig. 3:**
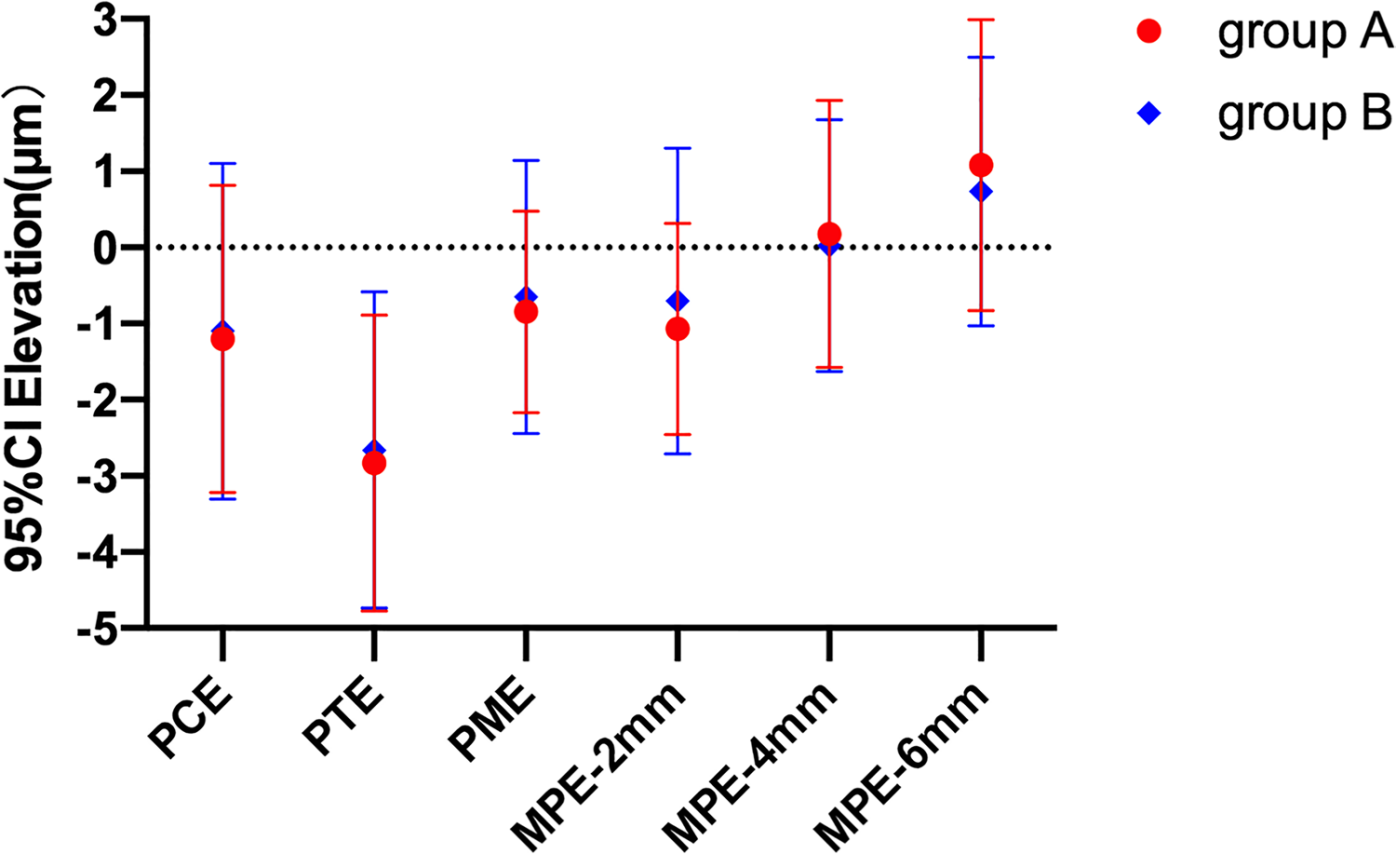
Changes in PCE, PTE, PME, MPE-2 mm, MPE-4 mm, and MPE-6 mm 3 years after SMILE. No statistically significant difference in either parameter was noted between the two groups (*P* > 0.05)

Eyes with a greater AR in group B had no statistically significant changes in all these determined points compared with those in group A (*P* ≥ 0.07). Changes in PCE, PTE, PME, and MPE-2 mm were negative numbers in both groups. However, MPE-4 mm and MPE-6 mm showed slight increased elevation in both groups. Detailed information on all changes is presented in Table 3. Figure 3 illustrates changes in posterior elevation, and different colors represent different groups.

**Table 3:** Changes of posterior corneal elevation at 3 years after SMILE in two groups

	PCE	PTE	PME	MPE-2 mm	MPE-4 mm	MPE-6 mm
Group A	− 1.14 ± 2.53	− 3.04 ± 1.49	− 0.87 ± 1.34	− 1.04 ± 1.75	0.21 ± 1.86	0.69 ± 1.95
Group B	− 1.06 ± 2.69	− 2.92 ± 1.63	− 0.72 ± 1.25	− 0.78 ± 1.96	0.11 ± 1.98	0.36 ± 1.81
P value	0.57	0.88	0.43	0.52	0.90	0.07

*PCE* posterior central elevation, *PTE* posterior thinnest elevation, *PME* posterior maximal elevation, *MPE*-2 mm mean posterior elevation in the central 2 mm region, *MPE*-4 mm mean posterior elevation in the central 4 mm region, *MPE*-6 mm mean posterior elevation in the central 6 mm region

^*^Difference is significant at the 0.05 level

### Correlation

The AR had no statistical relationship with changes in posterior elevation in either group. In group A, correlation tests indicated that changes in PCE, PTE, MPE-4 mm, and MPE-6 mm had no statistical relationships with preoperative spherical equivalent (SE), ablation depth (AD), residual bed thickness (RBT), cap thickness, or optical zone; a significant correlation was found between RBT and changes in PME and MPE-2 mm with *r* values of − 0.42 (*P* = 0.01) and − 0.40 (*P* = 0.01), respectively. In group B, no statistical relationship was observed between changes in posterior elevation and preoperative SE, AD, corneal cap thickness, or optical zone. Table 4 summarizes the comprehensive results of the correlation tests.

**Table 4:** Correlation between ablation ratio (AR), preoperative spherical equivalent (SE), ablation depth (AD), residual bed thickness (RBT), cap thickness (CAP), optical zone (OD), and the changes of posterior corneal elevation at 3 years after SMILE in two groups

		PCE		PTE		PME		MPE-2 mm		MPE-4 mm		MPE-6 mm	
		*r*	*P* value	*r*	*P* value	*r*	*P* value	*r*	*P* value	*r*	*P* value	*r*	*P* value
Group A	AR	− 0.14	0.41	0.01	0.99	− 0.15	0.37	− 0.16	0.32	− 0.13	0.43	0.08	0.64
	SE	− 0.17	0.31	0.06	0.74	0.02	0.92	0.19	0.26	0.28	0.09	0.02	0.92
	AD	− 0.19	0.25	− 0.09	0.58	0.13	0.44	0.30	0.07	0.12	0.47	− 0.14	0.41
	RBT	− 0.05	0.75	− 0.03	0.87	− 0.42	0.01*	− 0.40	0.01*	− 0.21	0.21	− 0.11	0.49
	CAP	− 0.13	0.45	− 0.08	0.65	0.26	0.11	0.27	0.10	0.14	0.38	− 0.27	0.12
	OD	− 0.20	0.23	0.01	0.96	0.17	0.31	0.25	0.13	0.23	0.16	− 0.10	0.55
Group B	AR	− 0.20	0.90	− 0.02	0.91	− 0.06	0.70	0.07	0.71	0.17	0.29	− 0.11	0.51
	SE	− 0.15	0.36	− 0.12	0.46	− 0.15	0.37	− 0.21	0.19	− 0.28	0.08	0.17	0.29
	AD	0.15	0.37	0.06	0.74	− 0.10	0.56	0.21	0.19	0.14	0.41	− 0.13	0.45
	RBT	0.17	0.31	− 0.06	0.72	− 0.08	0.61	0.18	0.26	− 0.01	0.96	0.01	0.97
	CAP	0.21	0.20	− 0.05	0.78	− 0.02	0.90	0.15	0.37	− 0.01	0.98	− 0.13	0.42
	OD	0.01	0.96	− 0.24	0.15	− 0.18	0.28	− 0.11	0.52	− 0.06	0.73	0.01	0.97

*AR* ablation ratio, *SE* preoperative spherical equivalent, *AD* ablation depth, *RBT* residual bed thickness, *CA* cap thickness, *OD* optical zone, *PCE* posterior central elevation, *PTE* posterior thinnest elevation, *PME* posterior maximal elevation, *MPE*-2 mm mean posterior elevation in the central 2 mm region, *MPE*-4 mm mean posterior elevation in the central 4 mm region, *MPE*-6 mm mean posterior elevation in the central 6 mm region

^*^Difference is significant at the 0.05 level

## Discussion

Ophthalmologists are attempting to avoid the incidence of iatrogenic keratectasia after corneal refractive surgery. The ablation ratio (AR), a relatively independent index combining corneal removal thickness and preoperative corneal thickness, is of great significance in surgical design and prediction of postoperative corneal stability [15]. The risk of iatrogenic keratectasia is elevated because of the removal of more corneal tissue in patients with high myopia than in those with moderate and low myopia [16]. Previous studies showed that excessive removal of the corneal tissue resulted in postoperative corneal protrusion after laser in situ keratomileuses (LASIK) [14, 17]. Compared with traditional excimer laser surgeries, SMILE is a less invasive procedure with a flapless, 2-mm small incision using femtosecond laser cutting. Because most anterior corneal collagen fibers are kept intact, SMILE may greatly maintain corneal mechanical integrity and is thus beneficial for postoperative corneal stability [18]. The current study specifically explored the stability of posterior corneal elevation after SMILE, as well as the correlation between AR and changes in posterior corneal elevation.

At the 3-year follow-up, our study showed that 98% of eyes achieved a UDVA of 20/20 or better. The safety and efficacy indexes in all groups were better than 1.00, and nearly half of the included eyes gained better CDVA postoperatively than preoperatively. Additionally, Pedersen et al. performed a 3-year follow-up of 87 high myopia eyes that underwent SMILE, and they found safety and effectiveness indexes of 1.13 and 0.91, respectively; among all the included eyes, 72% reached a UDVA of 20/20 or better [19]. Moreover, Xia et al. showed that 92.3% of high myopia eyes in the SMILE group had a UDVA better than or equal to 20/20, and eight eyes (10.3%) gained lines of CDVA [20]. The results suggest that SMILE has satisfactory safety and validity as a surgical alternative for myopia correction.

This study showed that the posterior corneal central area had a decreasing tendency at 3 years post SMILE, and some statistically significant differences were detected in both groups postoperatively compared with that preoperatively. In other cohorts of patients who underwent SMILE, long-term backward displacement in the posterior corneal surface had been observed as well. For example, in eyes with myopia between − 3.0 D and − 9.0 D, the mean values of PCE preoperatively and 3 years after SMILE were 0.97 ± 2.29 µm and − 1.42 ± 3.48 µm, respectively; the average values of PTE were 2.69 ± 3.15 µm and 0.36 ± 4.13 µm, respectively [21]. Similar change patterns were also observed in myopia higher than − 10.0 D, and PCE, PTE, and PME displayed backward displacements with values of − 1.18 ± 3.06 µm, − 1.94 ± 3.21 µm, and − 0.47 ± 3.07 µm, respectively [13]. Moreover, another study investigated outcomes in suspicious corneas after SMILE and found that suspicions topography did not increase the postoperative risk, and these corneas had comparable decreasing posterior corneal elevation variations with those with normal topography images [22]. Nevertheless, negative posterior corneal elevation changes in all these studies were small, suggesting that although the detectable differences could be academic, their clinical value remains to be observed. It should not be ignored that the reduction in posterior elevation could be by chance or due to the measurement error (e.g., the BFS may change after SMILE).

Region-dependent changes were found in both groups; the central 2 mm area showed a subtle declining elevation values, and the peripheral area (4-mm and 6-mm areas) slightly increased. Consistently, previous studies showed that posterior corneal elevation decreased in the central optical zone but increased in the peripheral area after SMILE [23, 24]. In addition, similar findings were also noted in posterior corneal elevation changes after Sub-Bowman Keratomileusis and LASIK [25, 26]. Notably, the hyperopic shift model has been described to explain these phenomena: corneal refractive surgery removes central corneal lamella, leaving broken central stromal lamella and relaxation of the peripheral lamellae, and ultimately contributes to central corneal flattening and peripheral steepening [23].

Correlation analyses revealed that AR had no statistical relationship with changes in posterior elevation. In contrast, Li et al. demonstrated that AR was correlated with postoperative PCE and PTE, and the cut-off points for increased changes were 27.3% and 27.1%, respectively [27]. Moreover, Cao et al. analyzed risk factors affecting changes in posterior corneal elevation, and the findings revealed that for every 10% increase in AR, posterior corneal elevation exhibited an average forward displacement of 0.6–1.0 µm; the cut-off value of AR in their study was 26.9–28.3% [23]. The distribution of eyes with different AR could account for the discrepancy between our study and those aforementioned studies. In our study, more than 85% of the included eyes had AR of less than 27%: only 10 eyes in group B had AR of more than 27%, and one eye had AR of more than 28.3%. We further analyzed AR in eyes that underwent SMILE from Jan 2019 to Mar 2019 and found that more than 93.8% of eyes had AR of less than 27%, and only 0.4% of eyes had AR of more than 28%. The reason for the low AR in our patient database was that most patients with high myopia were inclined to choose an implantable Collamer lens rather than SMILE. The results suggest that AR within certain limits has no impact on the changes in posterior corneal elevation.

No statistical correlation was found between changes in PCE, PME, MPE-4 mm, or MPE-6 mm and RBT; although PME and MPE-2 mm were found negatively correlated with RBT in group A, the coefficient was relatively low (*r* =  − 0.42 for PME and *r* =  − 0.40 for MPE-2 mm). To date, whether there is a relationship between posterior corneal changes and RBT remains unclear. Some previous results are consistent with ours [21, 24]. Moreover, no statistically significant relationship was found in the eyes with suspicious tomographic images either [22]. Nonetheless, opposite results were also reported in other studies [12]. The criterion for RBT could be traced back to 1999, and Wang et al. proposed that RBT should not be less than 250 μm to reduce the incidence of ectasia after LASIK [16]. The criterion has not been updated and is still used in SMILE. Recently, accumulating evidence has shown that because the strong tension-resistance anterior cornea stroma is preserved and the corneal cap holds up greater tension over time, SMILE facilitates greater biomechanical stability than LASIK [18, 28]. Accordingly, Reinstein et al. pointed out that eye rubbing is the main factor for ecstatic development, standardized SMILE surgery may not lead to iatrogenic keratectasia, and safety limits of RBT could be adapted from 250 to 220 μm [3]; in their study, the minimum corneal thickness was 541 μm, the maximum lenticule thickness was 178 μm, and the RBT thickness was 220 μm. The above-mentioned study may not be generalizable and we still strongly recommend RBT should not be less than 250 μm.

This study has several limitations. Firstly, the relatively small sample size could affect the accuracy of the results and their potential application in clinical practice. Secondly, we were unable to give an exact answer if it is safe to perform SMILE in eyes with AR of more than 28%. As mentioned above, there was low AR distribution in this study. But in Reinstein et al.’s study, the AR was 32.9% and their finding is inconsistent with our results. Lastly, other parameters evaluating ectasia, such as K value and corneal thickness, were not studied in the current study. Thus, further studies with a larger sample size and participants with higher AR, which could rule out the effects of some factors, such as eye rubbing, are needed to explore the impacts of SMILE on posterior corneal elevation.

## Conclusion

Our findings indicate that SMILE is an effective and safe surgical approach for high myopia correction, with the relative stability of the posterior corneal surface at the follow-up of 3 years, and the AR within certain limits has no impact on changes in posterior corneal elevation.

## Abbreviations

AD: Ablation depth
AR: Ablation ratio
BFA: Best-fit-sphere
CDVA: Corrected distance visual acuity
LASIK: Laser in situ keratomileuses
MPE: Mean posterior elevation
PCE: Posterior central elevation
PME: Posterior maximal elevation
PTE: Posterior elevation at the preoperative thinnest point
RBT: Residual bed thickness
SE: Spherical equivalent
SMILE: Small incision lenticule extraction
UDVA: Uncorrected distance visual acuity
