# Correction to: Correlation between the ablation ratio and posterior corneal stability after small incision lenticule extraction for high myopia

**DOI:** 10.1007/s00417-023-06015-2

**Published:** 2023-03-02

**Authors:** Liyuan Yang, Shengtao Liu, Xingtao Zhou, Yu Zhao

**Affiliations:** 1 Department of Ophthalmology, Gansu Provincial Hospital, Lanzhou, Gansu China; 2 Department of Ophthalmology and Optometry, Eye and ENT Hospital, Fudan University, 83 Fenyang Road, Shanghai, 200031 People’s Republic of China; 3 NHC Key Laboratory of Myopia (Fudan University), Laboratory of Myopia, Chinese Academy of Medical Sciences, Shanghai, China; 4 Shanghai Research Center of Ophthalmology and Optometry, Shanghai, China

**Correction to: Graefe’s Archive for Clinical and Experimental Ophthalmology**

10.1007/s00417-023-05979-5

In the published version of this article, the given name of the second author has been misspelled.

The correct author name should be “**Shengtao Liu**”.

This is being corrected in this publication.

